# Supplementary material for: CB1R blockade unmasks TRPV1-mediated contextual fear generalization in female, but not male rats
Source: Neuropsychopharmacology. 2023 Jul 17;48(10):1500–8. doi: 10.1038/s41386-023-01650-z (PMC10425366; doi:10.1038/s41386-023-01650-z)
Supplement: Supplementary file 1 — Supplemental Material [file 41386_2023_1650_MOESM1_ESM.docx]

| **Figure**  **Table S1: comprehensive statistics results** | **Test** | **Effect/comparison** | **Result** | **p value** |
| --- | --- | --- | --- | --- |
| 1B | Chi-square | Drug effect | Chi sq = 2.2 | P=0.53 |
| 1D | 2-way ANOVA | Trial x drug interaction | F (18, 276) = 1.110 | P=0.3414 |
|  |  | Main effect of trial | F (5.088, 234.0) = 0.7592 | P=0.5822 |
|  |  | Main effect of drug | F (3, 46) = 0.3702 | P=0.7749 |
| 1E | Mixed-effects model | Trial x drug interaction | F (18, 118) = 0.8232 | P=0.6700 |
|  |  | Main effect of trial | F (4.006, 78.79) = 2.573 | P=0.04* |
|  |  | Main effect of drug | F (3, 118) = 2.734 | P=0.047* |
| 1F | 2-way ANOVA | Trial x drug interaction | F (18, 324) = 0.7494 | P=0.7586 |
|  |  | Main effect of trial | F (4.953, 267.5) = 1.124 | P=0.3476 |
|  |  | Main effect of drug | F (3, 54) = 0.6473 | P=0.5880 |
| 2A (FC-BL) | 1-way ANOVA | Main effect of drug | F (3, 47) = 1.461 | P=0.2372 |
| 2A (FC-Tones) | 2-way ANOVA | Trial x drug interaction | F (18, 282) = 1.247 | P=0.2228 |
|  |  | Main effect of trial | F (5.022, 236.0) = 56.40 | P<0.0001 |
|  |  | Main effect of drug | F (3, 47) = 3.913 | P=0.0142 |
|  | Dunnett’s post-hoc | VEH vs. AM |  | P=0.003** |
|  |  | VEH vs. CPZ |  | P=0.18 |
|  |  | VEH vs. AM+CPZ |  | P=0.006** |
| 2A (EX-BL) | 1-way ANOVA | Main effect of drug | F (3, 47) = 7.461 | P=0.0003*** |
|  | Dunnett’s | AM vs. VEH |  | P=0.0002*** |
|  | Dunnett’s | AM vs. CPZ |  | P=0.0015** |
|  | Dunnett’s | AM vs. AM+CPZ |  | P=0.0057** |
| 2A (EX-Tone Blocks) | 2-way ANOVA | Trial x drug interaction | F (27, 423) = 1.062 | P=0.3834 |
|  |  | Main effect of trial | F (9, 423) = 14.05 | P<0.0001 |
|  |  | Main effect of drug | F (3, 47) = 4.018 | P=0.0126 |
|  | Dunnett’s | VEH vs. AM |  | P=0.29 |
|  | Dunnett’s | VEH vs. CPZ |  | P=0.96 |
|  | Dunnett’s | VEH vs. AM+CPZ |  | P=0.04* |
| 2A (EXR-BL) | 1-way ANOVA | Main effect of drug | F (3, 47) = 5.437 | P=0.0027** |
|  | Dunnett’s | AM vs. VEH |  | P=0.0077** |
|  | Dunnett’s | AM vs. CPZ |  | P=0.0057** |
|  | Dunnett’s | AM vs. AM+CPZ |  | P=0.0041** |
| 2A (EXR-Tone Blocks) | 2-way ANOVA | Trial x drug interaction | F (12, 188) = 0.8944 | P=0.5538 |
|  |  | Main effect of trial | F (3.342, 157.1) = 4.792 | P=0.0022** |
|  |  | Main effect of drug | F (3, 47) = 5.243 | P=0.0033** |
|  | Dunnett’s | VEH vs. AM |  | P=0.02* |
|  | Dunnett’s | VEH vs. CPZ |  | P=0.98 |
|  | Dunnett’s | VEH vs. AM+CPZ |  | P=0.003** |
| 2B (FC-BL) | 1-way ANOVA | Main effect of drug | F (3, 17) = 1.821 | P=0.1816 |
| 2B (FC-Tones) | 2-way ANOVA | Trial x drug interaction | F (18, 102) = 1.469 | P=0.1172 |
|  |  | Main effect of trial | F (3.965, 67.40) = 2.755 | P=0.0353* |
|  |  | Main effect of drug | F (3, 17) = 11.43 | P=0.0002*** |
|  | Dunnett’s | VEH vs. AM |  | P=0.88 |
|  | Dunnett’s | VEH vs. CPZ |  | P=0.05 (under powered) |
|  | Dunnett’s | VEH vs. AM+CPZ |  | P=0.0003*** |
| 2B (EX-BL) | 1-way ANOVA | Main effect of drug | F (3, 17) = 3.413 | P=0.0414* |
|  | Dunnett’s | AM+CPZ vs. VEH |  | P=0.02*  All other comparisons ns |
| 2B (EX-Tones) | 2-way ANOVA | Trial x drug interaction | F (27, 153) = 1.187 | P=0.2548 |
|  |  | Main effect of trial | F (3.914, 66.54) = 5.469 | P=0.0008*** |
|  |  | Main effect of drug | F (3, 17) = 1.690 | P=0.2070 |
| 2B (EXR-BL) | 1-way ANOVA | Main effect of drug | F (3, 18) = 2.378 | P=0.1037 |
| 2B (EXR-Tones) | 2-way ANOVA | Trial x drug interaction | F (12, 72) = 0.8340 | P=0.6156 |
|  |  | Main effect of trial | F (2.664, 47.96) = 1.925 | P=0.1443 |
|  |  | Main effect of drug | F (3, 18) = 0.3280 | P=0.8051 |
| 2C (FC-BL) | 1-way ANOVA | Main effect of drug | F (3, 54) = 0.9465 | P=0.4247 |
| 2C (FC-Tones) | 2-way ANOVA | Trial x drug interaction | F (18, 324) = 1.001 | P=0.4577 |
|  |  | Main effect of trial | F (5.093, 275.0) = 41.46 | P<0.0001**** |
|  |  | Main effect of drug | F (3, 54) = 3.000 | P=0.0384* |
|  | Dunnett’s | VEH vs. AM |  | P=0.66 |
|  | Dunnett’s | VEH vs. CPZ |  | P=0.94 |
|  | Dunnett’s | VEH vs. AM+CPZ |  | P=0.8 |
| 2C (EX-BL) | 1-way ANOVA | Main effect of drug | F (3, 54) = 1.124 | P=0.3477 |
| 2C (EX-Tones) | 2-way ANOVA | Trial x drug interaction | F (27, 486) = 1.046 | P=0.4038 |
|  |  | Main effect of trial | F (4.427, 239.1) = 18.56 | P<0.0001**** |
|  |  | Main effect of drug | F (3, 54) = 0.5748 | P=0.6340 |
| 2C (EXR-BL) | 1-way ANOVA | Main effect of drug | F (3, 54) = 1.930 | P=0.1357 |
| 2C (EXR-Tones) | 2-way ANOVA | Trial x drug interaction | F (12, 216) = 0.9654 | P=0.4830 |
|  |  | Main effect of trial | F (3.211, 173.4) = 3.831 | P=0.0093** |
|  |  | Main effect of drug | F (3, 54) = 0.8436 | P=0.4760 |
| 3B | Unpaired t-test | Effect of conditioning | t=2.602, df=18 | P=0.018* |
|  | Unpaired t-test | Effect of conditioning | t=0.2164, df=18 | P=0.831 |
|  | Unpaired t-test | Effect of conditioning | t=0.6415, df=18 | P=0.529 |
| 3C | Unpaired t-test | Effect of conditioning | t=0.5926, df=28 | P=0.558 |
|  | Unpaired t-test | Effect of conditioning | t=1.563, df=26 | P=0.13 |
|  | Unpaired t-test | Effect of conditioning | t=2.411, df=27 | P=0.023* |
| 4C | 2-way ANOVA | Sex x experience interaction | F (2, 41) = 0.9034 | P=0.4131 |
|  |  | Main effect of sex | F (1, 41) = 2.633 | P=0.1124 |
|  |  | Main effect of experience | F (2, 41) = 0.7036 | P=0.5007 |
| 4D | 2-way ANOVA | Sex x experience interaction | F (2, 41) = 0.9351 | P=0.4007 |
|  |  | Main effect of sex | F (1, 41) = 2.813 | P=0.1011 |
|  |  | Main effect of experience | F (2, 41) = 0.4887 | P=0.6170 |
| 4E | 2-way ANOVA | Sex x experience interaction | F (2, 41) = 0.5078 | P=0.6055 |
|  |  | Main effect of sex | F (1, 41) = 1.371 | P=0.2483 |
|  |  | Main effect of experience | F (2, 41) = 0.2398 | P=0.7879 |
| 5H | 2-way ANOVA | Sex x labeling interaction | F (1, 24) = 0.1981 | P=0.6603 |
|  |  | Main effect of sex | F (1, 24) = 0.02454 | P=0.8768 |
|  |  | Main effect of labeling | F (1, 24) = 66.56 | P<0.0001**** |
| 5I | Welch’s t-test | Main effect of sex | t=2.332, df=15.92 | 0.0332* |

**Supplemental Methods**

Fear conditioning and extinction procedures: Behavioral testing occurred in two different contexts (A and B) inside Coulbourn Instruments operant chambers. Context A consisted of a chamber with a grid floor, back and side metal walls, clear Plexiglas front door and ceiling, and white light. Context A was cleaned with 70% ethanol between rats. Context B consisted of a white opaque plastic floor and curved walls and was cleaned with Virkon solution between rats. On days 1 and 2, animals were habituated to context A and B, respectively, for 10 min. Auditory fear conditioning (day 3) was performed in context A. After a 5-min acclimation (baseline) period, all rats were exposed to seven conditioning trials. Each conditioning trial involved a presentation of the conditioned stimulus (CS; 80 dB, 4 Hz tone) for 30 s, co-terminating with a 1 s unconditioned stimulus (US; 0.65 mA shock). Inter-trial interval (ITI) between two consecutive CS-US pairings was 3 min. After conditioning, each rat was returned to its home cage. On day 4, rats underwent extinction training in context B, which consisted of a 5 min baseline period followed by 20 CS presentations with an ITI of 2 min. On day 5, rats received an extinction retrieval session in context B that consisted of a 2-min acclimation period, followed by five conditioned stimuli with 2-min ITIs. An overhead, infrared digital camera allowed videotaping (30 frames per second) during behavioral procedures. We used Ethovision software (Noldus) to generate raw velocity data sheets from all video files. These data sheets were then fed to our custom Python program ScaredyRat, which calculated freezing, darting, and shock response data for each animal.

Inclusion criteria for tracing studies: To assess surgical targeting, 50 µm BLA-containing sections were collected for all animals using a Leica VT1000 S vibrating microtome. Sections were mounted on microscope slides, cover-slipped, and examined for accurate targeting of the BLA. Animals were excluded from analysis if the injection had hit another brain region (e.g. central amygdala or entorhinal cortex) or if the BLA was too damaged to assess. In total, 2 females and 3 males were removed from analysis. A representative image of a successful target is shown in Figure 5A as well as below.


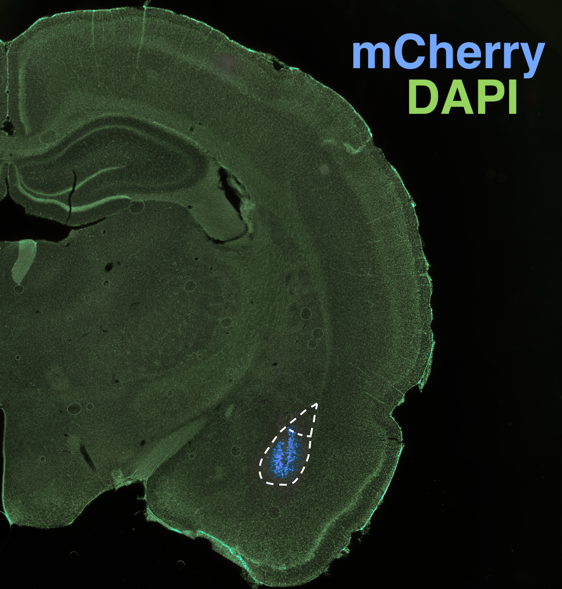


Ventral hippocampus CA1 identification and cell selection: The vHip boundaries were defined according to field standards [1,2] cross-referenced with our rat atlas [3]. The four sampled sections ranged on an anterior-posterior axis from Bregma -4.68mm to -6.48mm. No CA1 cells were included in analysis that were located outside the extent of observed retrograde labeling. To avoid unintentional bias in cell selection based on CB1 labeling intensity, both labeled and unlabeled cell targets were selected for imaging prior to switching to the 647 laser. To avoid potential confounds related to differences in antibody staining efficacy, photobleaching, or cell type variability, requirements for unlabeled cells selected for imaging were that cells a) must be at the same depth (Z) as the imaged labeled cell; b) must be in a neighboring but non-overlapping field of view; and c) must have a similar soma diameter as the imaged labeled cell. An example low magnification map of selected cells for one section is shown below. Numbers 1-4 correspond to the location of labeled cells selected for CB1 imaging; u1-u4 correspond to the location of unlabeled cells selected for imaging. B and B2 correspond to the location of unlabeled, unstained images collected and used for background fluorescence values.


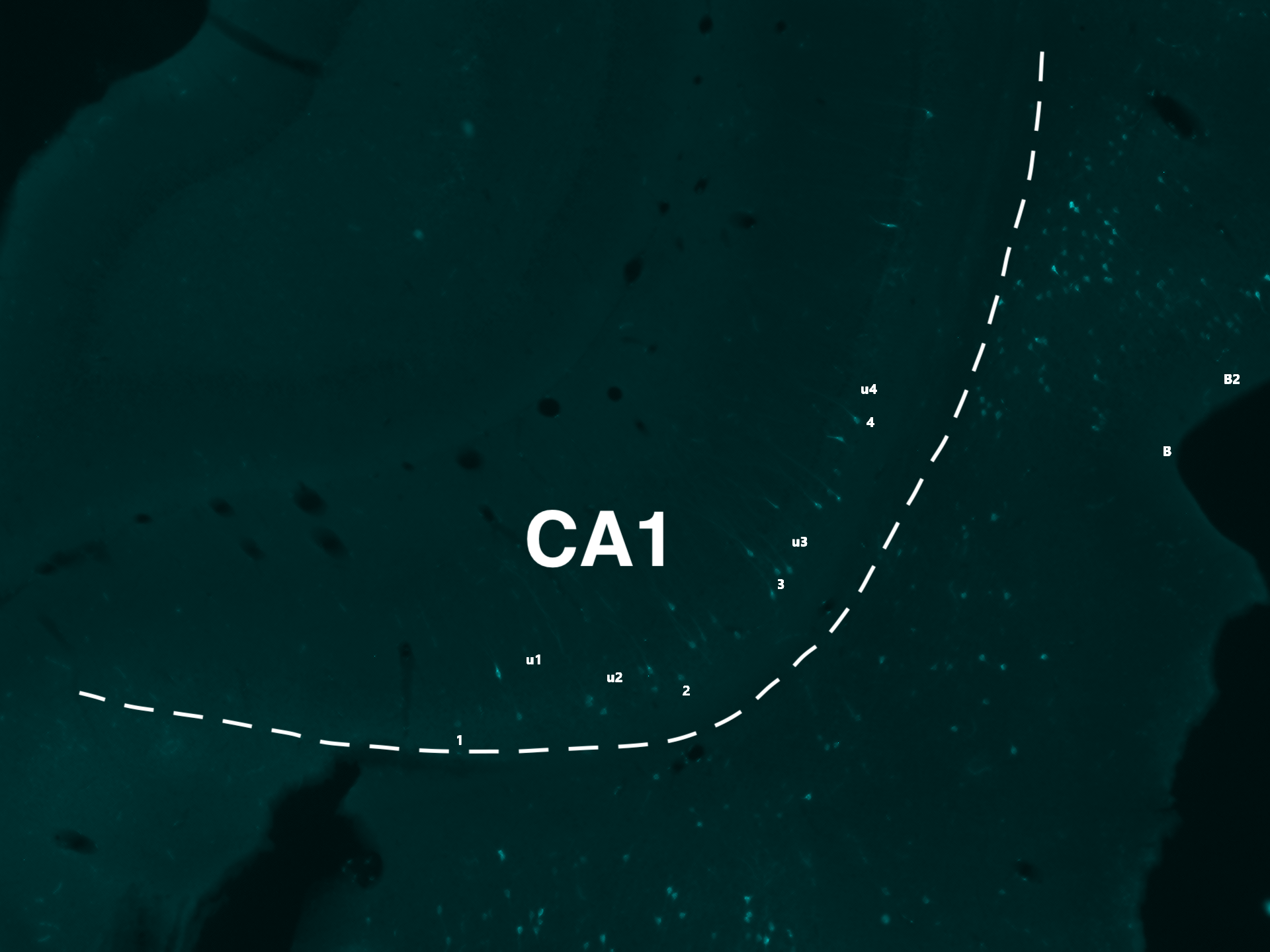


Supplemental Data: Freezing in CS+US animals included in Figure 3.

Sample images of CB1 staining around labeled and unlabeled cells.

The image below shows a pair of confocal images from the same animal, illustrating CB1 staining around a retrograde labeled (vHip-BLA) cell on the left vs. an unlabeled cell on the right. Location of the soma of each is shown in magenta.


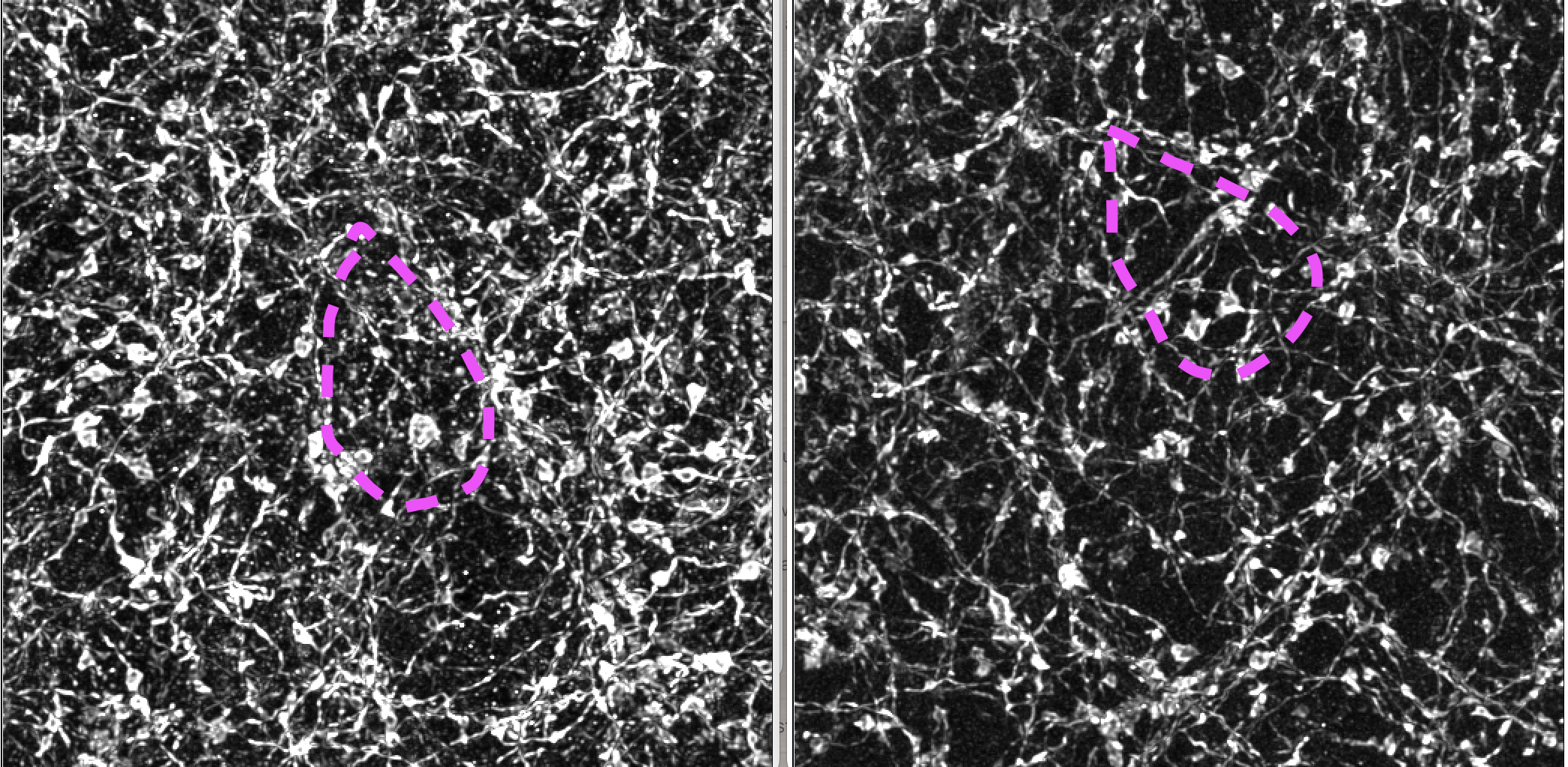


References

1. Swanson LW, Cowan WM. An autoradiographic study of the organization of the efferent connections of the hippocampal formation in the rat. J Comp Neurol. 1977;172:49–84.

2. Fanselow MS, Dong HW. Are the dorsal and ventral hippocampus functionally distinct structures? Neuron. 2010;65:7–19.

3. Paxinos G, Watson C. The Rat Brain in Stereotaxic Coordinates. 7th ed. Academic Press; 2013.
